# Effects of Atelocollagen Formulation Containing Oligonucleotide on Endothelial Permeability

**DOI:** 10.1155/2012/245835

**Published:** 2012-03-08

**Authors:** Koji Hanai, Takashi Kojima, Mika Ota, Jun Onodera, Norimasa Sawada

**Affiliations:** ^1^Formulation Research and Development Laboratories, Dainippon Sumitomo Pharma Co., Ltd., 1-3-45 Kurakakiuchi, Ibaraki-shi, Osaka 567-0878, Japan; ^2^Department of Pathology, Sapporo Medical University School of Medicine, S1, W17, Sapporo 060-8556, Japan; ^3^Environmental Health Science Laboratory, Sumitomo Chemical Co., Ltd., Osaka 554-0022, Japan; ^4^Research Institute, Koken Co., Ltd., 2-13-10, Ukima, Kita-ku, Tokyo 115-0051, Japan

## Abstract

Atelocollagen is a major animal protein that is used as a highly biocompatible biomaterial. To date, atelocollagen has been used as an effective drug delivery technology to sustain the release of antitumor proteins and to enhance the antitumor activity of oligonucleotides in *in vivo* models. However, the biological effects of this technology are not fully understood. In the present study, we investigated the effects of atelocollagen on endothelial paracellular barrier function. An atelocollagen formulation containing oligonucleotides specifically increased the permeability of two types of endothelial cells, and the change was dependent on the molecular size, structure of the oligonucleotides used and the concentrations of the oligonucleotide and atelocollagen in the formulation. An immunohistochemical examination revealed that the formulation had effects on the cellular skeleton and intercellular structure although it did not affect the expression of adherens junction or tight junction proteins. These changes were induced through p38 MAP kinase signaling. It is important to elucidate the biological functions of atelocollagen in order to be able to exploit its drug delivery properties.

## 1. Introduction

Collagen is a major connective tissue protein that plays an important role in the extracellular matrix in animals. As such, collagen possesses good biocompatibility with animal body tissues [1]. Atelocollagen is a type of soluble collagen produced from tropocollagen, the collagen molecule that makes up collagen fibrils, via the elimination of the telopeptide moieties, which are considered to account for most of collagen's antigenicity [1, 2]. Thus, atelocollagen is considered to have little immunogenicity, which makes it a safe biomaterial [1]. In fact, it is widely used for implantable medical and plastic surgical products [1].

Atelocollagen is also used as a drug delivery carrier. For example, a minipellet atelocollagen formulation has been demonstrated to sustain the release and maintain stable blood concentrations of protein drugs for more than 1 week [2]. Many kinds of protein drugs such as interferon-*α* [3], interleukin-2 [4], nerve growth factor [5], and bone morphogenetic protein [6], and so forth, have been administered using this drug delivery system, and interferon-*α* and interleukin-2 showed strong antitumor activities in animal models when administered in this manner [3, 4]. In the past decade, as well as being used as a solid substrate, dissolved atelocollagen has been used as a drug delivery vehicle for nucleic acid-based medicines for gene conversion [7], inflammatory disease [8, 9], and tumor therapy. Atelocollagen can be used to deliver most kinds of nucleic acid-based medicines including plasmid DNA [10], antisense oligodeoxynucleotides (ODN) [11–13], short interference RNA (siRNA) [14–20], and micro RNA (miRNA) [21–23]. It is also capable of delivering oligonucleotides to subcutaneous xenografts and metastatic tumors after its local and/or systemic administration.

Many studies, including some involving *in vivo* tumor models, have evidenced the contribution of atelocollagen to the enhancement of drugs' antitumor activities, and some of them described the mechanisms. For example, nucleic acids delivered by atelocollagen are protected against degradation by host nucleases [8, 14, 24], and it has also been shown to improve the delivery efficiency of oligonucleotides to tumors [15, 16]. However, the biological functions of atelocollagen and the mechanism by which it enhances delivery efficiency are still not fully understood. It is essential to reveal the biological characteristics of atelocollagen in order to be able to fully exploit its drug delivery potential. While we were studying the basic properties of atelocollagen, we discovered another of its functions: it increases endothelial permeability. Here, we describe the results of a study of the effects of atelocollagen on intercellular sealing function. We measured transendothelial electrical residence (TER) in order to estimate intercellular barrier function and performed an immunohistochemical examination to see whether any cellular morphological changes were induced.

## 2. Materials and Methods

### 2.1. Atelocollagen, Oligonucleotides, and Formulations

Atelocollagen was supplied in aqueous form by Koken (Tokyo, Japan). Rhodamine red-conjugated atelocollagen was prepared in accordance with the manufacturer's instructions (FluoReporter Rhodamine Red-X Protein Labeling Kit; Life technologies Japan, Tokyo, Japan).

The oligodeoxynucleotides (ODN) and double stranded RNA (dsRNA) were synthesized by Eurogentec (Seraing, Belgium). The sequences of the oligonucleotides are listed in Table 1 [20, 25, 26].

Each atelocollagen-oligonucleotide formulation (AC formulation) was prepared by gently mixing aqueous atelocollagen with a solution containing a defined concentration of oligonucleotides. The final oligonucleotide concentration was usually 5 *μ*M and that of atelocollagen was 0.1% w/v unless otherwise stated in the text, tables, and/or figures.

### 2.2. Transendothelial Electrical Resistance (TER) Measurement

Normal human dermal microvascular endothelial cells (HMVEC) were purchased from EIDIA (Tokyo, Japan). These cells were cultured in EGM (Endothelial Growth Media; EIDIA, Tokyo, Japan) until they reached confluence on 12 mm transwell filters with a 0.4 *μ*m pore size (Corning Glass Works; Corning Japan, Tokyo, Japan) coated with rat tail collagen. Porcine brain microvascular endothelial cells (BMVEC) were purified and maintained according to the method described in a previous study [27].

TER was determined using an EVOM voltohmmeter and an ENDOHM-12 chamber (World Precision Instruments, Sarasota, FL) at 37°C [28]. Cell growth was monitored by measuring TER. Once stable intercellular seals had formed; that is, at confluence, the medium in the inner chamber was exchanged for 400 microliters of culture medium containing 30% v/v of AC formulation. TER was subsequently measured in duplicate every 30 min.

### 2.3. Measurement of Paracellular Flux

HMVEC cells were also used for the determination of paracellular flux. One hour after treatment with the AC formulation, which was performed as described in Section 2.2, 100 *μ*L of 0.36% w/v Texas red-conjugated dextran (MW: 40 kDa; Life Technologies Japan, Tokyo, Japan) were added to the inner chamber. One hour later, the dextran concentration of the medium in the outer chamber was analyzed by measuring its fluorescence.

To investigate the enhancement of paracellular transport by atelocollagen, solute transportation was compared among the AC formulation, bovine serum albumin, and dextran. Once stable intercellular seals had formed, the medium in the inner chamber was exchanged for 400 microliters of culture medium containing 30% v/v of the AC formulation, which had been produced using 0.1 or 0.3% w/v rhodamine red-conjugated atelocollagen (i.e., approximately 0.03 or 0.1% w/v atelocollagen was added; molecular weight (MW): 300 kDa); 0.1% w/v of fluorescein conjugated dextran (MW: 70 kDa; Life Technologies Japan, Tokyo, Japan); 0.1% w/v of Alexa Fluor 594 conjugated bovine serum albumin (BSA; MW: 66 kDa; Life Technologies Japan, Tokyo, Japan). The solute concentrations of the outer chambers were analyzed by measuring their fluorescence at 1 and 2 hours after the medium exchange.

### 2.4. Immunohistochemistry

After treatment for 1 hr with the AC formulation, oligonucleotide alone, atelocollagen alone, or phosphate-buffered saline (PBS) as a control, HMVEC cells were fixed with 1% paraformaldehyde for 10 min and then treated with 0.2% Triton X-100 for 10 min. After preincubation with 5% skimmed milk, they were incubated for 1 hr at room temperature with rabbit or mouse antibodies against vascular endothelial (VE)-cadherin (BD Biosciences, San Diego, CA), zonula occludens-1 (ZO-1) (Zymed Laboratories, San Francisco, CA), claudin-5 (Zymed Laboratories, San Francisco, CA), and *α*-tubulin (Amersham, Poole, UK). Then, the samples were incubated for 1 hr with appropriate secondary antibodies labeled with Alexa Fluor-488 or Alexa Fluor-596 (Life Technologies Japan, Tokyo, Japan). Actin filaments were labeled with Alexa Fluor-546 phalloidin (Life Technologies Japan, Tokyo, Japan). The cells were thoroughly rinsed with PBS between each procedure. The expression of each protein was examined using a laser scanning confocal microscope (MRC 1024; Bio-Rad, Hercules, CA).

### 2.5. Western Immunoblotting

Western blotting was performed according to the method described in a previous report [29]. For Western blotting of the total cell lysates, the dishes were washed with PBS, and then 300 *μ*L of sample buffer (1 mM NaHCO_3_ and 2 mM phenylmethylsulfonyl fluoride) was added to 60 mm culture dishes. The cells were scraped and collected in microcentrifuge tubes and then sonicated for 10 sec. The protein concentrations of the samples were determined using a BCA (bicinchoninic acid) protein assay reagent kit (Pierce Chemical, Rockford, IL). For each sample, aliquots of protein (15 *μ*g per lane) were separated by electrophoresis in 4/20% sodium dodecyl sulfate polyacrylamide gels (Cosmo Bio, Tokyo, Japan) (SDS PAGE). After being electrophoretically transferred to nitrocellulose membranes (Immobilon; Millipore, Billerica, MA), the membranes were saturated with blocking buffer (trisbuffered saline [TBS] supplemented with 0.1% tween 20 and 4% skimmed milk) for 30 min at room temperature and incubated with antiactin, anti-ZO-1, anti-VE-cadherin (BD Biosciences, San Diego, CA), anticlaudin-5 (Zymed Laboratories, San Francisco, CA), anti-p38 mitogen-activated protein kinase (MAP Kinase or MAPK) (Santa Cruz Biotechnology, Santa Cruz, CA), antiphospho-p38 MAPK (Cell Signaling, Beverly, MA), anti-p42/44 MAPK (Promega, Madison, WI), antiphospho-p42/44 MAPK (Cell Signaling, Beverly, MA), anti-Rho-A, and anti-cdc42 (Santa Cruz Biotechnology, Santa Cruz, CA) antibodies (1:1000) for 1 h at room temperature. The membranes were then incubated with horseradish peroxidase-conjugated anti-rabbit or mouse IgG (Dako A/S, Copenhagen, Denmark) at room temperature for 1 h. The immunoreactive bands were detected using an ECL Western blotting analysis system (GE Healthcare, Little Chalfont, UK).

## 3. Results

### 3.1. Modification of Endothelial Sealing Function

**Figure 1:**
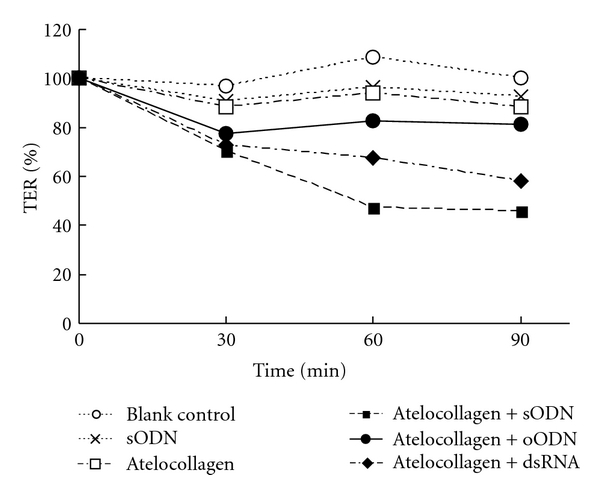
Time-dependent reduction of TER after treatment with different types of oligonucleic acids in combination with atelocollagen. HMVEC cells were treated with 5 *μ*M of oligonucleic acids with or without 0.1% atelocollagen. sODN: phosphorothioate oligodeoxynucleotide, oODN: phosphodiester oligodeoxynucleotide, dsRNA: small double stranded RNA. The sequences of the ODN are described in Table 1(a).

Paracellular flux is dependent on the function of tight junctions [30, 31]. We assessed the effects of an AC formulation on the TER of HMVEC to evaluate their tight junction function. As shown in Figure 1 and Table 1(a), the ODN containing AC formulation caused a time-dependent reduction in TER, while TER was hardly affected by treatment with ODN or atelocollagen alone. As for the type of oligonucleotide in the formulation, phosphorothioate ODN produced a more significant reduction in TER than phosphodiester ODN, which only produced slight alterations. A change in the TER value was also induced by treatment with small dsRNA.

Various formulations containing different ratios of ODN and atelocollagen were examined in order to understand which parameters have the greatest effect on the change in TER. As a result, we found that the TER change was dependent on the size of the ODN and the composition of the formulation, but not the ODN sequence, as shown in Tables 1(b), 1(c), and 1(d). Specifically, ODN composed of 15 or more bases were effective and those containing around 30 bases were the most effective, but 10-base-long ODN were not effective. The change in tight junction function was also dependent on the concentrations of ODN and atelocollagen in the formulation.

**Figure 2:**
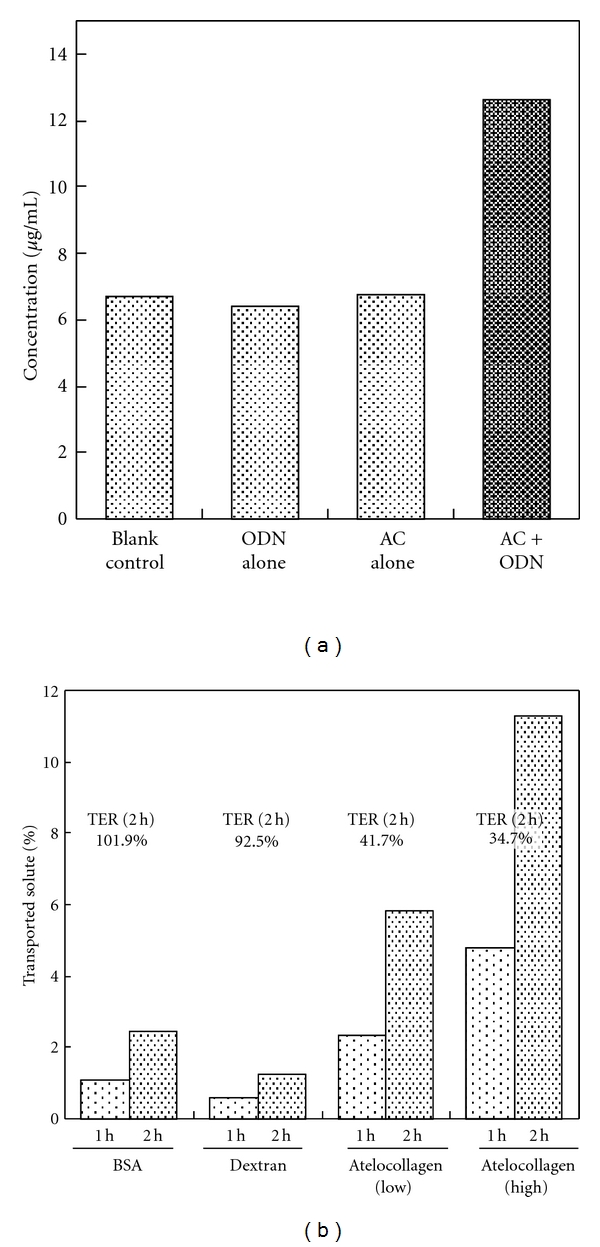
Effects of the AC formulation on endothelial paracellular flux. (a) Increase in dextran permeation induced by the AC formulation. One hour after the addition of oligodeoxynucleotides (ODN), atelocollagen (AC), or the AC formulation (AC + ODN) to the inner chambers, Texas red-conjugated dextran (MW: 40,000) was added to the inner chambers. One hour later, the dextran concentration of the medium in the outer chambers was determined. (b) Enhanced paracellular transport of atelocollagen. 0.1% w/v of Alexa Fluor 594 conjugated bovine serum albumin (BSA; MW: 66 kDa), 0.1% w/v of fluorescein conjugated dextran (dextran; MW: 70 kDa), or rhodamine red-conjugated atelocollagen (0.03% w/v: atelocollagen (Low); 0.1 % w/v: atelocollagen (High); MW of atelocollagen: 300 kDa) with 5 *μ*M of ODN was added to the inner chambers. The solute concentrations of the outer chambers were analyzed by measuring their fluorescence at 1 and 2 hours after the start of the experiment. TER at 2 hours is represented as a percentage value compared to that observed at the start of the experiment.

To verify that the AC formulation increased paracellular flux, the amount of Texas red-labeled dextran (molecular weight: 40 kD) transported across an endothelial cell layer was examined. As shown in Figure 2(a), dextran transport was increased approximately twofold in the cell cultures incubated with the AC formulation.

Next, the paracellular transport of atelocollagen was analyzed and compared with those of BSA and dextran. Neither BSA nor dextran affected the TER value of the cells. Only very small amounts of BSA and dextran penetrated the cell sheet during the 2-hour study period; on the other hand, much more atelocollagen passed through, even though the molecular weight of atelocollagen is 4-5 times higher than those of BSA and dextran (Figure 2(b)).

**Figure 3:**
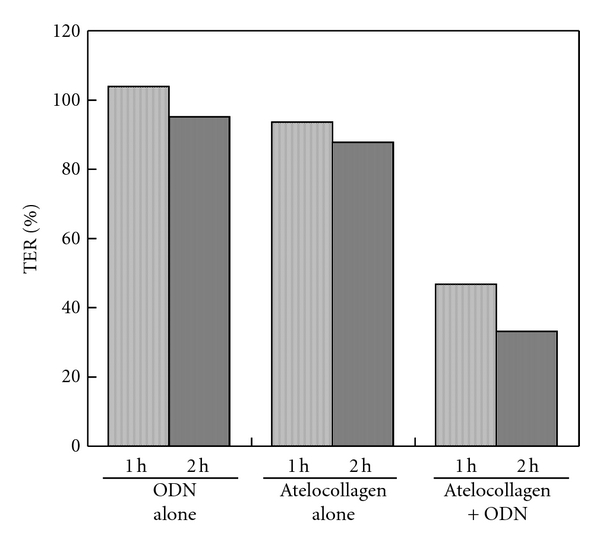
Effects of the AC formulation on the TER of BMVEC. The bar represents TER as a percentage compared to the value observed at the start of the experiment. TER was determined at 1 and 2 hours after treatment with ODN alone (ODN), atelocollagen alone (atelocollagen), the AC formulation (atelocollagen + ODN).

An examination using BMVEC [27] was performed to determine whether the effect of the AC formulation was specific to HMVEC. As a result, we found that the TER value of the BMVEC was also reduced by the AC formulation (and only the AC formulation), as shown in Figure 3. BMVEC forms the blood-brain barrier (BBB), where intercellular sealing function is strictly maintained. These results showed that the AC formulation is able to affect the paracellular flux of endothelial barriers.

### 3.2. Effects on Cell Morphology

**Figure 4:**
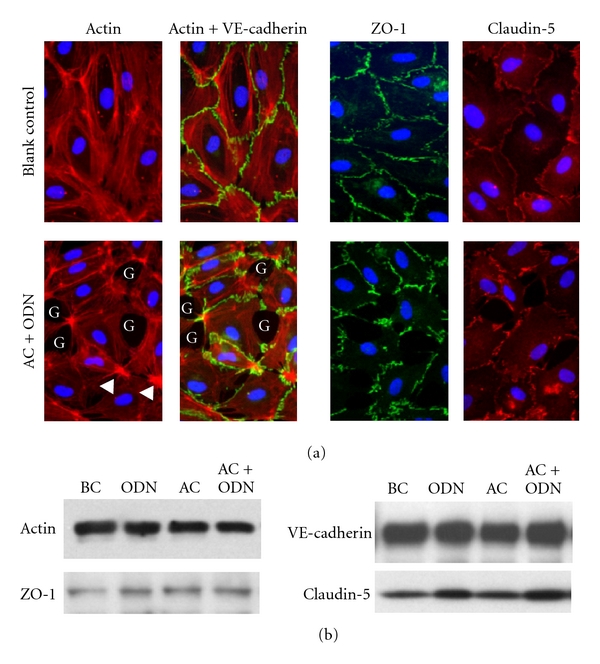
Effects of atelocollagen combined with ODN on intercellular formation. (a) Images of actin, VE-cadherin with actin, ZO-1, and claudin-5 in the endothelial cells of the blank control (upper panels) and the cells treated with the AC formulation (lower panels). Treatment with the AC formulation (AC + ODN) induced changes in cell morphology associated with stress fiber formation (arrowhead), intercellular gap formation (G), and the loss of VE-cadherin and ZO-1. (b) Western blot analysis of actin, VE-cadherin, ZO-1, and claudin-5. The whole cell extract obtained from the cells was separated by SDS-PAGE and immunoblotted with the corresponding antibodies. BC: blank control, ODN: treated with ODN alone, AC: treated with atelocollagen alone, AC + ODN: treated with the AC formulation.

It is well known that increased endothelial permeability is associated with impaired intercellular contact [32–35]. We carried out an immunohistochemical analysis of the cells treated with the AC formulation to clarify how their intercellular sealing was affected. As shown in Figure 4(a), treatment with the AC formulation markedly reduced the degree of intercellular contact, as shown by intercellular gap formation, actin stress fiber formation, cellular contraction, and a lack of VE-cadherin. Adequate expression of claudin-5, one of the key components of the endothelial barrier, was noted at the cell periphery. However, ZO-1 protein expression was absent from the intercellular gaps. On the contrary, Western blotting revealed that treatment with the AC formulation did not affect the expression of these proteins (Figure 4(b)). Although the TER value remained low as long as the AC formulation was present in the culture medium, the treatment did not cause toxicity. The cells survived well for at least 24 hrs, and both the TER and morphology of the cells could be recovered by removing the formulation (data not shown). No such morphological changes were induced by treatment with ODN or atelocollagen alone (Figure 4(a)).

**Figure 5:**
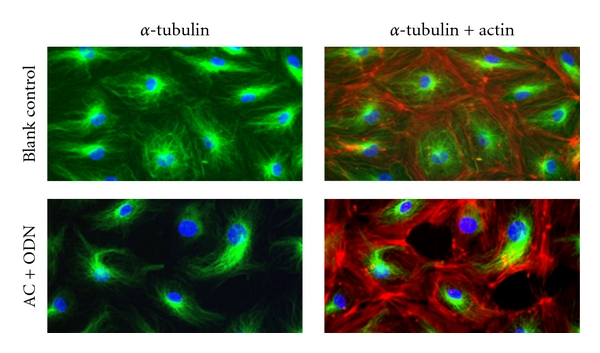
Effects of the AC formulation on the microtubule networks in the endothelial cells. Images of *α*-tubulin (left panels) and *α*-tubulin (green) with actin (red) (right panels). In the blank control (upper), the microtubule network was composed of extended fine microtubule structures. After treatment with the AC formulation (AC + ODN), the fine microtubules had been disassembled, and the network had disintegrated (lower).

Microtubules play an important role in regulating actin formation and hence, endothelial barrier function [36, 37]. We also histopathologically examined the shape of the microtubules. As shown in Figure 5, *α*-tubulin formed a fine network in the blank control. However, treatment with the AC formulation caused the peripheral fine structure of the *α*-tubulin network to be lost.

### 3.3. Activation of Signal Transduction-Related Molecules

**Figure 6:**
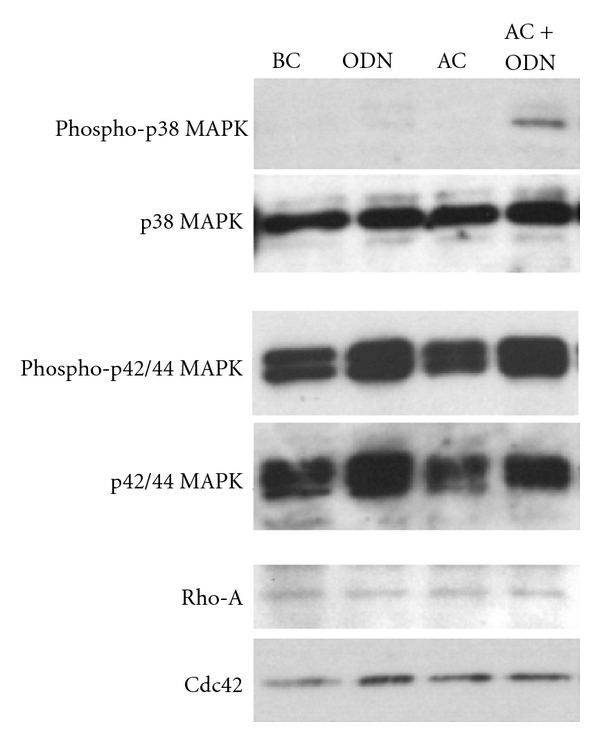
Effects of the AC formulation on the expression of signal transduction-related molecules. Western blot analysis of the expression levels of p38 MAP-kinase, the p42/44 MAP-kinases, and their phosphorylated forms, RhoA and Cdc42 was performed after each treatment. BC: blank control, ODN: treated with ODN alone, AC: treated with atelocollagen alone, AC + ODN: treated with AC formulation. p38 MAP-kinase activation was only enhanced by the AC formulation.

Many studies have shown that increased endothelial permeability and impaired intercellular contact can be induced by signal transduction, mainly that of Rho A [32, 38] and p38 MAP kinase [36, 39–42]. Thus, we investigated the effects of the AC formulation on signal transduction. As a result, no differences were found in the expression levels of Rho-A, Cdc42, or P42/44 MAP kinases or their phosphorylated forms. Regarding p38 MAP kinase, although no changes were noted among the control, oligonucleotide alone, or atelocollagen alone groups, the levels of phosphorylated p38 MAP kinase were markedly increased in the cells treated with the AC formulation (Figure 6), which indicates that the impact of the AC formulation on tissue permeability is associated with the activation of p38 MAP kinase.

## 4. Discussion

Collagen plays an important role in the extracellular matrix by supporting cells so that they can form tissues and organs. Atelocollagen is produced from type I collagen and is widely used in its solid state as a biomaterial for medical and surgical products because of its biocompatibility and workability [1]. However, the kinetics, dynamics, and biological functions of atelocollagen after its injection into the living body are still poorly understood, and it is essential to elucidate the characteristics of atelocollagen in order to fully exploit its potential.

Here, we demonstrated a novel biological function of atelocollagen. When endothelial cell sheets were treated with atelocollagen or oligonucleotides alone, the intercellular structure of the sheet was not changed. However, when the atelocollagen and oligonucleotides were administered together, intercellular gaps formed and consequently the paracellular flux of the sheet was elevated. The AC formulation itself was also able to penetrate the sheet. This function might explain the ability of atelocollagen to effectively deliver oligonucleotides. The abovementioned changes were elicited via the activation of p38 MAP kinase, a signal-transduction-related molecule. In addition, the changes observed in this study are similar to those triggered by thrombin [32], histamine [33], TNF-*α* [34, 36], and VEGF (vascular endothelial growth factor) [43], and so forth. As shown in Section 3.1, the degree to which endothelial function was affected was dependent on the molecular structure of the oligonucleotides including their size and chemical modifications, suggesting that the three-dimensional structure of the oligonucleotide and atelocollagen complex stimulates a signal transduction pathway that acts as a permeability modulator, although the specific pathway that it stimulates remains unknown.

To date, no severe systemic edema or side effects of the AC formulation have been noted, even after the intravenous administration of atelocollagen as an oligonucleotide drug carrier. These findings indicate that atelocollagen could be used as a permeability enhancer at local treatment sites without the adverse systemic effects that cytokines and chemokines sometimes provoke. Since tight junction modulators are regarded as practical drug delivery enhancer candidates [44–46], the function of atelocollagen demonstrated in the present study should be thoroughly investigated.

 The unique biological functions of atelocollagen have led to the development of unique antitumor therapies and products, such as surgical products; formulations that sustain the release of antitumor proteins [2–4]; treatments that enhance the antitumor activities of various molecules including antisense ODN [11–13], siRNA [14–20, 24], and miRNA [21–23]. Obtaining more information about atelocollagen would allow us to develop the next generation of atelocollagen-mediated drug delivery systems.

## Figures and Tables

**Table 1a:** (a) Effects of oligonucleotide type.

Oligonucleotides				Atelocollagen concentration	TER (%)
Type, sequences		Reference	Concentration		
Blank control					100.0
Phosphorothioate Oligodeoxynucleotide	5′-TGCATCCCCCAGGCCACCAT-3′	25	5 *μ*M		92.7
Atelocollagen alone				0.1%	88.1
Phosphorothioate Oligodeoxynucleotide	5′-TGCATCCCCCAGGCCACCAT-3′	25	5 *μ*M	0.1%	45.7
Phosphodiester Oligodeoxynucleotide	5′-TGCATCCCCCAGGCCACCAT-3′		5 *μ*M	0.1%	81.4
Double stranded small RNA	5′-UGCAUCCCCCAGGCCACCAUdTdT-3′ and complemental sequence	20	5 *μ*M	0.1%	57.9

**Table 1b:** (b) Effects of ODN sequence on TER (%).

Oligonucleotides				Atelocollagen	
Name	Sequence	Reference	Concentration	0%	0.1%
				TER (%)	
Blank control				100	—
ODN-(1)	5′-TGCATCCCCCAGGCCACCAT-3′	25	5 *μ*M	100	58
ODN-(2)	5′-TCGCATCGACCCGCCCACTA-3′	25	5 *μ*M	100	54
ODN-(3)	5′-GCTGATTAGAGAGAGGTCCC-3′	26	5 *μ*M	100	50
ODN-(4)	5′-CCCTGGAGAGAGATTAGTCG-3′		5 *μ*M	96	65

**Table 1c:** (c) Effects of ODN length on TER (%).

Oligonucleotides		Atelocollagen	
Sequence	Concentration	0%	0.1%
Examination (1)		TER (%)	
Blank control	—	94.6	—
Atelocollagen alone	—	—	94.6
ODN 10-mer (5′-TGCATCCCCC-3′)	5 *μ*M	106.3	102.8
ODN 15-mer (5′-TGCATCCCCCAGGCC-3′)	5 *μ*M	94.1	47.1
ODN 20-mer (5′-TGCATCCCCCAGGCCACCAT-3′; Ref. [25])	5 *μ*M	107.1	50.0
ODN 30-mer (20-mer ODN + 10-mer ODN)	5 *μ*M	102.9	43.8

Examination (2)		TER (%)	
Blank control	—	87.4	—
Atelocollagen alone	—	—	83.3
ODN 20-mer (5′-TGCATCCCCCAGGCCACCAT-3′; Ref. [25])	5 *μ*M	91.7	51.4
ODN 30-mer (20-mer ODN + 10-mer ODN)	5 *μ*M	83.3	26.5
ODN 50-mer (20-mer ODN × 2 + 10-mer ODN)	5 *μ*M	85.7	35.3
ODN 100-mer (20-mer ODN × 5)	5 *μ*M	82.4	50.0

**Table 1d:** (d) Effects of the concentrations of ODN and atelocollagen on TER (%).

ODN concentration	Atelocollagen			
	0%	0.003%	0.03%	0.1%
	TER (%)			
Blank control	94.6			
0.5 *μ*M	100.0			77.8
5 *μ*M	102.8			43.7
50 *μ*M	102.9			50.8
500 *μ*M	91.7			28.5

	TER (%)			
Atelocollagen alone				100.0
5 *μ*M	94.9	98.1	54.9	52.0

ODN sequence: 5′-TGCATCCCCCAGGCCACCAT-3′.

TER(%): relative value at 1.5 hrs compared to the value observed at the start of the experiment.
